# The effect of dental chair light exposure duration on shear bond strength of orthodontic brackets: An *in vitro* study

**DOI:** 10.4317/jced.55296

**Published:** 2018-11-01

**Authors:** Kevin M. Andrews, Glen Roberson, Karthikeyan Subramani, Kishore Chaudhry

**Affiliations:** 1 Roseman University of Health Sciences, College of Dental Medicine, Henderson, NV, USA

## Abstract

**Background:**

The purpose of this study is to determine if the duration of exposure to the halogen overhead dental chair light has an effect on shear bond strength (SBS) of metal orthodontic brackets.

**Material and Methods:**

One hundred twenty extracted human lower incisor teeth were divided into six groups (n=20/group). Each group was assigned a predetermined duration of exposure to the halogen dental chair light, set at a fixed distance, before being cured. Light exposure times of 0 minutes (Group 1-Control), 1 minute (Group 2), 2.5 minutes (Group 3), 5 minutes (Group 4), 10 minutes (Group 5), and 15 minutes (Group 6) were tested. Each tooth was subjected to an exclusion criteria examination, scrubbed of all debris, and imbedded in a PVC-stone fixture with the crown of the tooth exposed above the stone surface. All groups had orthodontic brackets bonded with the same materials and process, then light cured for 6 seconds using the Valo LED curing unit after their designated light exposure time. Groups were tested using an Instron E-1000 universal testing machine with a shear load test set at a speed of 1mm/min using a knife-edged chisel. Data was analyzed using a one-way ANOVA test. The Adhesive Remnant Index (ARI) was scored under 10x magnification. The ARI data was analyzed using the Chi-square test (*p*-value < 0.05).

**Results:**

All control and experimental groups for each specific tooth type tested resulted in SBS within or above the clinically acceptable range. Statistically significant differences (*p*<.05) were found between the control and experimental groups for dental chair light exposure times of 5 minutes, 10 minutes and 15 minutes. A chi-square test determined that there was statistical significance when evaluating the frequency of ARI scores when light exposure duration was greater than 5 minutes.

**Conclusions:**

It can be concluded that dental chair light exposure in the 5 minute, 10 minute and 15 minute groups produced higher shear bond strength than those of the control, 1 minute and 2.5 minute groups. The dental chair light is capable of initiating polymerization and causing higher bond strengths than the clinical acceptability of 5.8-7.9 MPa, thus continued dental chair light exposure over 5 minutes is not recommended. The ARI analysis revealed that as bond strength increased, the fracture pattern shifted from most remaining adhesive attached to the tooth toward that attached to the bracket.

** Key words:**Shear bond strength, orthodontic bracket, adhesive remnant index, dental chair light, light exposure, composite curing.

## Introduction

The fixation of orthodontic brackets to the surface of teeth is a frequently utilized procedure in every orthodontic office. Light polymerization, or light curing, is needed in order to physically adhere the bracket to the enamel surface by initiation of a chemical reaction in which the adhesive is changed from a paste to a hardened resin (1). The polymerization is initiated through the use of visible light in the blue range of the electromagnetic spectrum to excite the outermost layer of the camphoroquinone that possesses an absorption spectrum between 400 and 500 nm, being most efficient in the 468 to 470 nm range (2).

There are many choices of light curing sources for the photopolymerization of the adhesive in orthodontics such as halogen, plasma arc, argon laser, and light-emitting diode units (LED). However, only two light sources are used in the dental chair operator light, halogen and LED. Newer models of overhead dental chair lights with LED have settings that use the yellow spectrum of light instead of blue during the curing process. This is an important factor to prevent premature curing of the dental composite. In a previous study conducted by Dlugokinski *et al.*, ambient light, which was classified as environmental light present in the room, had minimal to no effect on the curing of the composite resin (3). The author also found that when introducing light emitted from the overhead dental chair light for durations of 2, 5 and 10 minutes, curing of the composite occurred at 60%, 73% and 78% of max cure respectively (3).

Tiwari *et al.* (2) tested effects of the dental chair operator light on shear bond strength, and found that with increasing light exposure, there was an increase in shear bond strength. However, this study did not take into account the duration the samples were exposed to the operator light.

The purpose of this *in vitro* study was to investigate the effect of the overhead dental chair light exposure time on the shear bond strength (SBS) of light cured composite resin.

## Material and Methods

-Test Samples

**Figure 1:**
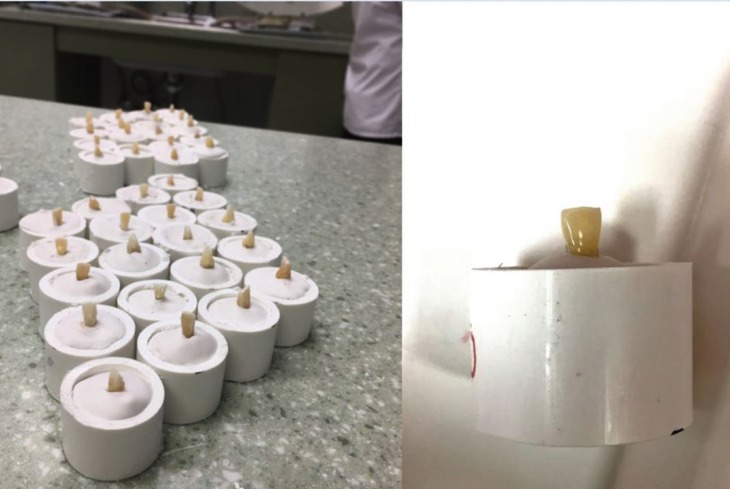
Mounted samples.

**Figure 2:**
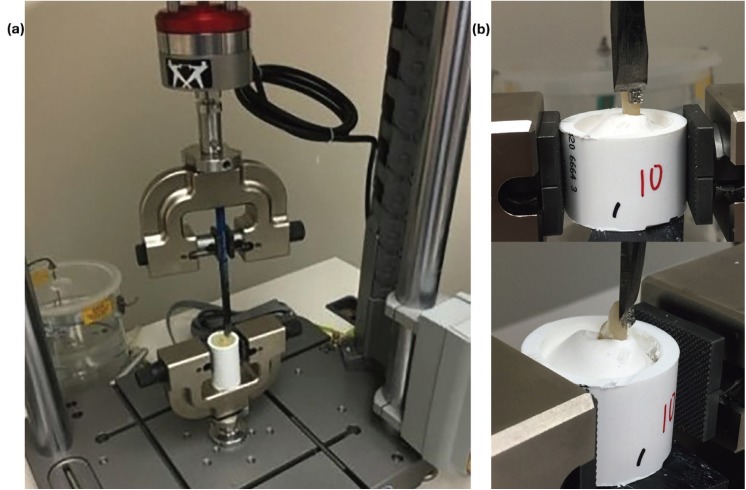
a) Instron testing machine with sample held in position. b) Instron attachment blade placed at the bracket ligature groove ready for testing at a crosshead speed of 1mm/min.

One hundred twenty extracted human mandibular incisors with intact buccal surfaces were collected from various sources. Teeth were stored in a 1:100 sodium hypochlorite (Clorox, Oakland, CA) solution from the time of collection to the time of bonding.4 Teeth were mounted in 1 inch diameter PVC pipe using Type I Dental Stone (Snow White #2-Kerr Corporation., Orange, CA) with the long axis perpendicular to the floor and parallel to shearing attachment of the Instron Electropuls E1000 Universal testing machine (Illinois Tool Works Inc., Norwood, MA) (Fig. 1,2a).

-Exclusion Criteria

The teeth were subjected to a specific exclusion criteria protocol. Teeth with hypoplastic enamel, visible cracks, gross irregularities in enamel structure, caries, extrinsic stains, or restorations on the bonding surface were excluded from the study.

-Brackets and Bonding Materials

One hundred twenty Dentsply GAC Standard Twin Medium mandibular incisor metal orthodontic brackets (Islandia, NY) with a bracket base area of 11.92 mm² were used in this study. The etchant used was Opal Etch 35% phosphoric acid (Ultradent, South Jordan, UT). Assure Plus Universal Bonding Resin and Light Bond adhesive (Reliance Orthodontic Products, Inc., Itasca, IL) were used to bond the brackets to the extracted teeth.

-Bonding Procedure

Each tooth was subjected to 10 seconds of prophylactic treatment with a non-fluoride containing pumice (Henry Schein, Melville, NY), water rinse and air dried.5 Opal etch (35% phosphoric acid) was applied to the enamel surface and left in place for 15 seconds, then rinsed thoroughly for 20 seconds until all etchant was removed. The enamel surface was dried with oil and moisture-free air until a chalky appearance was observed (6,7). A thin layer of Assure Plus bonding primer was applied to the etched enamel surface and was subsequently air dried for 1-2 seconds and light cured for two seconds (7). A thin layer of Light Bond resin paste, was uniformly applied to the mandibular lower incisor bracket base. The bracket was then positioned in the center of the prepared enamel surface, both mesio-distally and inciso-gingivally, with the bracket oriented in line with the long axis of the tooth. It was then pressed firmly using a Dontrix gauge (American Orthodontics, Sheboygan, WI) with approximately 300g of force. Any excess adhesive was removed using a dental scaling instrument without dislodging the bracket.

The teeth were divided into six groups (n=20/group), according to the bonding protocol as follows:

Each sample was light cured using the Valo light emitting diode curing unit (Ultradent, South Jordan, UT) at 3,200 mW/cm² for 6 seconds total (3 seconds on the mesial and 3 seconds on the distal) after the designated overhead dental chair light exposure time. Batteries in the curing lamp were changed every 10 samples and curing unit power was tested with a light intensity sensor to ensure consistent light power every 5 samples.

Control Group (Group 1): With the overhead dental chair light placed in the OFF position, the bracket was positioned on the center of the tooth with 300g of force and with archwire slot perpendicular to long axis of the tooth. Flash was carefully removed and the bracket was light cured for 3 seconds on each of the mesial and distal aspect of the bracket (6 seconds total) using a Valo LED curing unit at 3,200 mW/cm² at a distance of 2 mm from the bracket-tooth interface.

Experimental Groups (Groups 2-6): With the overhead dental chair light placed in the ON position and the light positioned at 30 inches above the sample, the Group 2 bracket was positioned on the center of the tooth with 300g of force and with the archwire slot perpendicular to long axis of the tooth. Flash was carefully removed and sample was left exposed to dental chair light for a total of one minute before the bracket was cured using the Valo LED curing light. All subsequent groups were conducted in the same fashion within their respective groups. Group 3 was allowed a continued exposure to the dental chair light for 2.5 minutes, Group 4 for an overall exposure time of 5 minutes, and Group 5 for 10 minutes of exposure. Group 6 had 15 minutes of continued dental chair light exposure prior to being cured.

After bonding and curing, the samples were arranged on a tray and covered with a moist towel. They were then placed in an opaque packing box after being covered completely by another opaque tray to prevent other sources of light from additionally curing the composite material.

-Testing Procedure

All samples were tested on an Instron Electropuls E1000 Universal testing machine (Illinois Tool Works Inc., Norwood, MA). The archwire slot was positioned parallel to the horizontal plane. A knife-edged chisel was placed in contact with the incisal portion of the bracket base between the bracket pad and tie wings (ligature groove) parallel to the long axis of the tooth, creating a shearing force in the inciso-gingival direction (Fig. 2).8,9 The specimen was subjected to a compressive load at a crosshead speed of 1.0 mm/min until failure.10,11 The force in Newtons (N) was recorded for each sample at the time of bracket debond.

-Adhesive Remnant Index

The debonded bracket was observed under 10x magnification with a macro lens. Photographs were taken of each bracket to score the adhesive remnant index (ARI). ARI was recorded for each bracket at two separate time points two weeks apart. ARI was scored according to the following grading system: (12)

0: 100% of the adhesive remaining on the bracket

1: More than 50% of the adhesive remaining on the bracket

2: Less than 50% of the adhesive remaining on the bracket

3: No adhesive remaining on the bracket

-Shear Bond Strength (SBS) Calculation and Statistical Analysis

Shear bond strength was calculated using the following formula:

SBS (MPa) = Force (N)/Surface area of bracket base (mm2) (13). Mean and standard deviation of SBS values were calculated using SPSS version 25 (IBM Chicago, IL, USA). A One-way ANOVA test was used for comparisons of SBS between multiple groups with *p*-value of 0.05 or less being considered as statistically significant. The ARI scores were compared using a chi-square analysis to determine if there was a statistically significant difference among the dental chair light time exposure groups.

## Results

**Figure 3:**
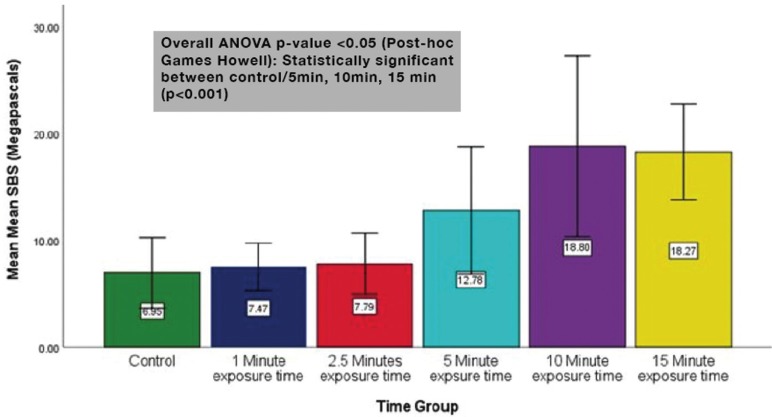
Mean Shear Bond Strength with Standard Deviation ±2.

Results for the shear bond strength values are shown in  Table 1 and Fig. 3. A One-way ANOVA was used to determine statistical significance. The teeth bonded in the control group (Group 1) with no exposure to overhead dental chair light showed the lowest SBS of 6.95 ±2.69 Megapascals (MPa). The subsequent groups displayed increasing SBS corresponding to the increase in time of exposure to the dental chair light. The 1 minute dental chair light exposure group (Group 2) had a mean SBS of 7.47 ±1.05 MPa and was found to be not statically significant (*p*=0.999) when compared to the control group. Group 3 (2.5 minutes of exposure) had a mean SBS of 7.49 ±1.77 MPa and was found not to be statically significant with (*p*=0.999) when compared to the control group. Groups 1, 2 and 3 had shear bond strengths that all fell within the range of clinically acceptable range of 5.8 – 7.9 MPa as reported by Reynolds (13). The mean of the remaining groups 4, 5, and 6 exceeded the clinically acceptable shear bond strengths, while groups 5 and 6 approached hazardous levels as reported by Dall’Igna *et al.* (14) Group 4 (5 minutes of exposure) was found to have a statistically significant higher SBS (*p*<0.05) than the control group with a mean of 12.78 ± 5.43 MPa. Groups 5 and 6 were also found to have statistically significant higher (*p* <0.001) SBS of 18.80 ± 6.78 MPa and 18.27 ± 4.49 MPa respectively, as compared with the control group.

**Table 1:**
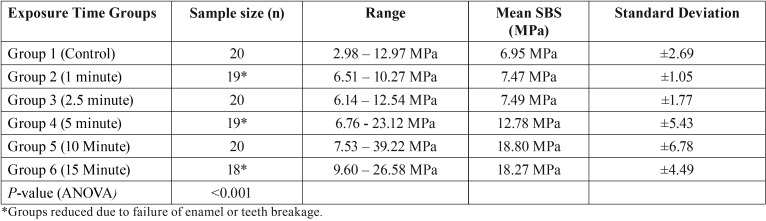
Mean shear bond strength in megapascals (MPa). The results are shown in Mean and Standard Deviation.

**Figure 4:**
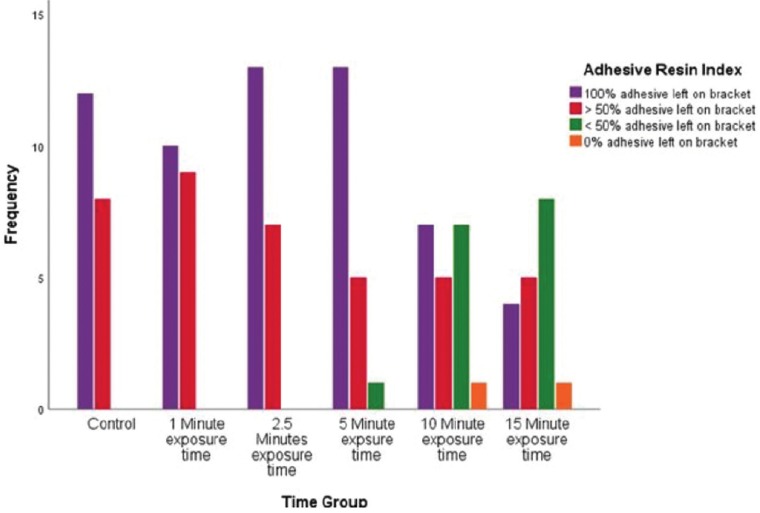
ARI Frequency Distribution Chart.

The results of the ARI analysis are shown in  Table 2, Fig 4. The groups were separated by dental chair light exposure time and failure pattern. Chi-square analysis showed a statically significant difference (*p*< 0.001) with increased exposure time and bond failure pattern among groups. The control and experimental groups 2 and 3 had predominant scores of 0 and 1. As the time of exposure of the overhead dental light increased in groups 4, 5 and 6, the ARI score shifted to a more even distribution between 0-2.

**Table 2:**
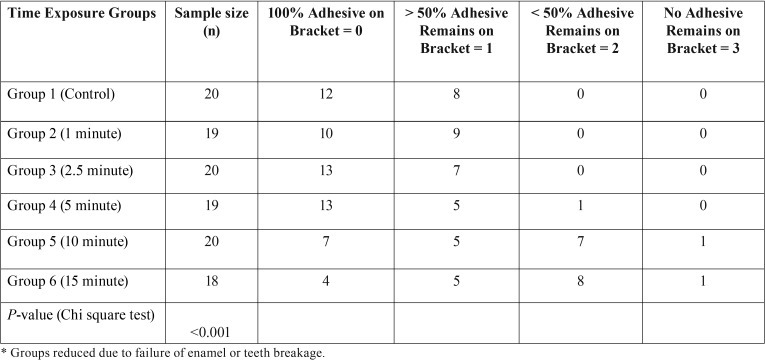
Adhesive remnant index.

## Discussion

During this *in-vitro* study, all controllable variables were kept consistent throughout the entire study. Although all variability cannot be controlled or accounted for, factors such as specimen storage, mounting procedures, bracket type, bonding protocol and materials, overhead dental chair light distance, LED light curing unit batteries, and LED intensity were kept constant during the entirety of the testing. By standardizing all controllable variables, only the shear bond strength would be altered by the increased light exposure time from the dental chair. Factors that could not be accounted for included using extracted teeth from multiple sources, storage of those teeth until used in the study, length that extracted teeth were stored until testing, and dehydration of the teeth during both the mounting phase and bonding phase. Four samples were discarded due to fracture or complete breakage of the tooth while conducting the testing. Statistics were calculated using only the successful samples.

Orthodontic bond failure has long been a cause of concern for the clinician, resulting in loss of continuity of patient care, increased treatment length, and decreased profit for the orthodontist (15). Thus it is critical to achieve at least the clinically acceptable shear bond strength of 5.8-7.9 MPa as reported by Reynolds (13). To do this, the proper amount of light energy is needed in order to cause sufficient polymerization of the composite material to achieve the necessary bond strength. This study evaluated the relationship of the addition of the dental chair light and its effect on the shear bond strength of the bracketed tooth. A total of 120 teeth were tested, consisting of 6 groups of 20 samples per group. The control group showed a mean of 6.95 ±2.69 MPa with a range of 2.98 – 12.97 MPa. This was consistent with a previous study conducted by Tiwari *et al.* with a control range of 5.74 ±1.13 MPa. This was well within the range of clinically acceptable shear bond strength as stated by Reynolds (2,13).

Sample Group 2 (1 minute of exposure) and Group 3 (2.5 minutes of exposure) resulted in similar mean SBS of 7.47 ±1.05 MPa and 7.49 ±1.77 MPa, respectively. These results were also consistent with those found by Tiwari *et al.*, which resulted in shear bond strengths of 7.71 ±1.90 MPa after dental chair light exposure (2). This confirms the previous results, that exposure to the dental chair light does increase shear bond strength.

The remaining experimental groups showed a sharp increase in shear bond strength that far exceeded the previous study. Group 4 (5 minutes of exposure) had a mean SBS of 12.78 ±5.43 MPa which is well above the clinical standard and above the previous study. Group 5 (10 minutes of exposure) and Group 6 (15 minutes of exposure) proved to have the highest SBS values of 18.80 ±6.78 MPa and 18.27 ±4.49 MPa, respectively. These values approach what was reported in the literature by Dall’Igna *et al.* to be dangerous (14). He stated that as SBS approaches 20.0 MPa, samples would start to fracture. Dangerous levels of SBS by excessive polymerization could result in unwanted enamel fracture when brackets are removed from a patient’s teeth. In that some samples in the control group showed SBS below the clinically acceptable range and many samples exposed to chair light for 5 or more minutes showed very high SBS, it may be appropriate to conclude that exposure to dental chair light should be limited to 1 to 2.5 minutes.

The adhesive remnant index scores of this study used visual inspection of the dislodged orthodontic brackets to provide an analysis of where the failure occurred. The ARI scoring used for the present study was based on a scale of 0-3 as reported by Oz *et al.* (12). In the present study, as well as the previous study conducted by Tiwari *et al.*, the control and experimental groups 2 and 3 had predominant scores of 0’s and 1’s with the fracture occurring at or within the composite-bracket interface (2). Interestingly, as the SBS increased above the clinically acceptable levels, the fracture pattern shifted toward more failures occurring at the composite-tooth interface. ARI scores patterns were found to be statistically significant in this study (*p* <.001) which was contradictory to the previous study conducted by Tiwari.

## Conclusions

This study demonstrated that the duration of exposure to the overhead dental chair light does cause increased shear bond strength of the orthodontic bracket. Thus, confirming the previous study by Tiwari *et al.*, indicating that the dental chair light is capable of initiating composite polymerization (2). It can also be concluded that an excessive amount of light energy during the bonding process by means of delayed curing, can produce dangerous SBS levels. It should be advised that patients should not be exposed to more than 5 minutes with the dental chair light in the on position during bond procedures. The ARI analysis revealed that the adhesive fracture pattern does change as light exposure is prolonged.
